# Ethanol and Caffeine Effects on Social Interaction and Recognition in Mice: Involvement of Adenosine A_2A_ and A_1_ Receptors

**DOI:** 10.3389/fnbeh.2016.00206

**Published:** 2016-11-02

**Authors:** Laura López-Cruz, Noemí San-Miguel, Pilar Bayarri, Younis Baqi, Christa E. Müller, John D. Salamone, Mercé Correa

**Affiliations:** ^1^Àrea de Psicobiologia, Campus de Riu Sec, Universitat Jaume ICastelló, Spain; ^2^Pharma-Zentrum Bonn, Pharmazeutisches Institut, Pharmazeutische Chemie, Universität BonnBonn, Germany; ^3^Department of Psychological Sciences, University of ConnecticutStorrs, CT, USA

**Keywords:** ethanol, adenosine, social exploration, social memory, anxiety, caffeine

## Abstract

Ethanol and caffeine are frequently consumed in combination and have opposite effects on the adenosine system: ethanol metabolism leads to an increase in adenosine levels, while caffeine is a non-selective adenosine A_1_/A_2A_ receptor antagonist. These receptors are highly expressed in striatum and olfactory tubercle, brain areas involved in exploration and social interaction in rodents. Ethanol modulates social interaction processes, but the role of adenosine in social behavior is still poorly understood. The present work was undertaken to study the impact of ethanol, caffeine and their combination on social behavior, and to explore the involvement of A_1_ and A_2A_ receptors on those actions. Male CD1 mice were evaluated in a social interaction three-chamber paradigm, for preference of conspecific vs. object, and also for long-term recognition memory of familiar vs. novel conspecific. Ethanol showed a biphasic effect, with low doses (0.25 g/kg) increasing social contact and higher doses (1.0–1.5 g/kg) reducing social interaction. However, no dose changed social preference; mice always spent more time sniffing the conspecific than the object, independently of the ethanol dose. Ethanol, even at doses that did not change social exploration, produced amnestic effects on social recognition the following day. Caffeine reduced social contact (15.0–60.0 mg/kg), and even blocked social preference at higher doses (30.0–60.0 mg/kg). The A_1_ antagonist Cyclopentyltheophylline (CPT; 3–9 mg/kg) did not modify social contact or preference on its own, and the A_2A_ antagonist MSX-3 (1.5–6 mg/kg) increased social interaction at all doses. Ethanol at intermediate doses (0.5–1.0 g/kg) was able to reverse the reduction in social exploration induced by caffeine (15.0–30.0 mg/kg). Although there was no interaction between ethanol and CPT or MSX-3 on social exploration in the first day, MSX-3 blocked the amnestic effects of ethanol observed on the following day. Thus, ethanol impairs the formation of social memories, and A_2A_ adenosine antagonists can prevent the amnestic effects of ethanol, so that animals can recognize familiar conspecifics. On the other hand, ethanol can counteract the social withdrawal induced by caffeine, a non-selective adenosine A_1_/A_2A_ receptor antagonist. These results show the complex set of interactions between ethanol and caffeine, some of which could be the result of the opposing effects they have in modulating the adenosine system.

## Introduction

Alcohol and caffeine are the most consumed psychoactive drugs worldwide. In recent times, it has become common to consume high doses of caffeine in combination with ethanol in order to reduce the intoxicating effects of the alcohol (Ferré and O’Brien, 2011; López-Cruz et al., 2013; Correa et al., 2014). Caffeine and ethanol act on the adenosine system in distinct ways that can result in opposite physiological and behavioral effects. Caffeine is a non-selective adenosine antagonist that acts mainly on A_1_ and A_2A_ receptors (Fredholm et al., 1999), whereas ethanol has been demonstrated to increase the adenosinergic tone by inhibiting the endonucleotid transporter type-1, thus, blocking adenosine uptake (Nagy et al., 1990; Krauss et al., 1993), and also by increasing the synthesis of adenosine during ethanol metabolism (Carmichael et al., 1991; López-Cruz et al., 2013).

Adenosine is a neuromodulator in the central nervous system (CNS) that plays an important role in the regulation of synaptic transmission and neuronal excitability (Cunha, 2001; Sebastião and Ribeiro, 2009). Several subtypes of adenosine receptors are expressed in the brain, with A_1_ and A_2A_ being the most abundant. A_2A_ receptors are expressed at high levels, mostly in the striatum and olfactory bulbs and tubercle (Schiffmann et al., 1991; Fredholm et al., 2001), regions that are involved in the regulation of motivated (Salamone and Correa, 2002, 2012; Hauber and Sommer, 2009), and social behaviors (Sano et al., 2008; Pena et al., 2014). However, A_1_ receptors have a widespread distribution in the brain, with a somewhat higher concentration in hippocampus (Schwarzschild et al., 2006).

It is well known that ethanol consumption facilitates interactions with peers and alleviates anxiety (Varlinskaya and Spear, 2002; Kirchner et al., 2006). In rodent models of social interaction, acute ethanol administration at low doses produces social facilitation (Nadal et al., 1993; Varlinskaya and Spear, 2009), but dose-related decrements in social interaction after high doses also have been observed in mice (Lister and Hilakivi, 1988; Hilakivi et al., 1989). Caffeine was shown to decrease social interaction in mice and rats (Baldwin and File, 1989; Baldwin et al., 1989; Hilakivi et al., 1989), effects that have been suggested to be related to its anxiogenic actions (Baldwin et al., 1989; Hilakivi et al., 1989; Prediger et al., 2004). However, very little is known about the interaction of both substances on social exploration and social memory (Hilakivi et al., 1989; Spinetta et al., 2008). The amnestic effect of ethanol is well known. Although ethanol at low doses can act as a short-term social memory enhancer in mice (Manrique et al., 2005), high doses of ethanol can cause amnesia, or impaired retrieval of memory, after the drug wears off (Goodwin, 1995; Hartzler and Fromme, 2003). This effect of ethanol could be explained by the fact that adenosine and adenosine receptor agonists have been demonstrated to impair short-term social recognition memory in rats (Prediger and Takahashi, 2005). On the other hand, selective A_1_ and A_2A_ receptor antagonists can improve short-term social memory (Prediger and Takahashi, 2005).

The present work evaluated the effect of a broad range of doses of caffeine, in combination with ethanol, on social motivation as measured by preference towards a conspecific vs. a neutral object. Our procedure minimized anxiety induced by aggression, avoiding whole-body contact. In a second phase of the behavioral test, long-term social recognition memory was studied 24 h after the drug was administered and the preference test had taken place. In addition, the role of A_1_ and A_2A_ receptors on social motivation and memory were also evaluated using selective adenosine antagonists alone or in combination with ethanol.

## Materials and Methods

### Subjects

Adult male CD1 mice (30–45 g) were purchased from Janvier (France). Mice were housed in groups of three per cage, with standard laboratory rodent chow and tap water available *ad libitum*. They were maintained in the colony at 22 ± 1°C with lights on from 8:00 to 20:00 h. All experimental procedures were approved by “Comité de bienestar animal, UJI” and complied with the European Community Council directive (86/609/ECC) for the use of laboratory animal subjects and with the “Guidelines for the Care and Use of Mammals in Neuroscience and Behavioral Research” (National Research Council 2003).

### Drugs

Caffeine (Sigma-Aldrich, Spain) and MSX3 ((*E*)-phosphoric acid mono-[3-[8-[2-(3-methoxphenyl)vinyl]-7-methyl-2,6-dioxo-1-prop-2-ynyl-1,2,6,7-tetrahydropurin-3-yl] propyl] ester disodium salt; synthesized at the laboratory of Dr. Christa E. Müller at the Pharmazeutisches Institut, Universität Bonn, Germany) were dissolved in 0.9% w/v saline. 8-cyclopentyltheophylline (CPT; purchased from Sigma-Aldrich, Spain) was dissolved in distilled water (pH = 8.0). All these drugs were administered intraperitoneally (IP) 30 min before testing. Ethanol (Panreac Quimica S.A., Spain) was diluted to 20% (v/v) in physiological saline (0.9% w/v) and administered IP 10 min before testing. Saline solution was used as vehicle. These doses and time leads were selected based on previous studies done in our laboratory with the same strain of mice (Correa et al., 2008; Pardo et al., 2013; López-Cruz et al., 2014). For the interaction studies we selected doses that did not impair locomotion, but showed some effect in the social procedures. The dose of CPT was selected because it was the one closest to reaching a significant effect in the social interaction test.

### Behavioral Apparatus and Testing Procedures

#### Social Preference and Social Recognition Tests

**Figure 1:**
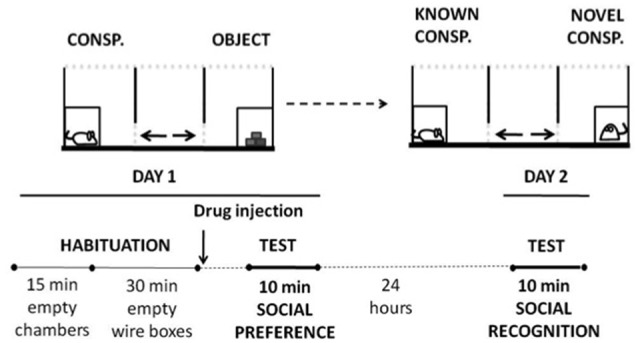
**Schematic representation of social preference and social recognition tests settings and timeline**.

The effects of adenosine antagonists on social preference were measured in a three-chambered social box (originally developed by Crawley, 2004). The general procedure was adapted from Chévere-Torres et al. (2012). Every mouse had two consecutive habituation sessions in the chambers: in the first one, they freely explored the empty social arena during 15 min, and immediately there was a second exploration session, that lasted 30 min, in the presence of two wire cages, one in each of the side-compartments. After the 45-min habituation period, different groups of animals received their corresponding treatment and were placed in an individual cage during 10 or 30 min (depending on the drug). After this time, mice were placed in the center chamber of the social interaction apparatus and test started. During the test session (10 min), the three-chambered arena contained a caged conspecific on one side, and on the other side there was a small wire cage containing an object. The center compartment was empty (see Figure 1 for a schematic on the procedure). The placement of the conspecific or the object was counterbalanced between animals. A trained experimenter who was unaware of the experimental conditions, registered manually time spent sniffing each target (conspecific vs. object) as a measure of social preference. Vertical and horizontal locomotion were also registered. Twenty-four hours after the social preference test, mice were placed back in the central chamber and were subjected to a 10 min social recognition test (Moy et al., 2004). No drugs were administered before this second test. During the recognition test a novel mouse replaced the object, and the experimental mice were given the choice to interact with the familiar conspecific (same conspecific used in the social preference test the day before) vs. a novel conspecific. Time spent sniffing each conspecific was registered.

### Statistics

One-way analysis of variance (ANOVA) was used to analyze the effect of drug administration on the different dependent variables; time spent sniffing conspecific, object, familiar and novel conspecific, and vertical and horizontal locomotion. Two-way factorial ANOVA was used for the interaction studies. When the overall ANOVA was significant, non-orthogonal planned comparisons using the overall error term were used to compare each treatment with the control group (Keppel, 1991). For these comparisons, α level was kept at 0.05 because the number of comparisons was restricted to the number of treatments minus one. Student’s *t*-test for dependent samples was used to analyze “preference” (e.g., conspecific vs. object, or familiar vs. novel conspecifics). A probability level of 0.05 or smaller was used to indicate statistical significance. Statistics were done using STATISTICA 7 software.

## Results

### Experiment 1: Effect of Ethanol on Social Preference and Locomotion: Impact on Long-Term Social Recognition Memory

**Figure 2:**
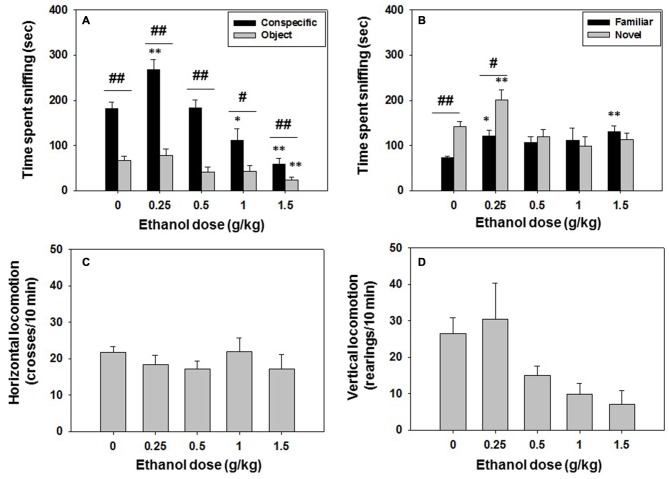
**Effect of ethanol in social preference and recognition tests.** Data are expressed as mean (±SEM) of time spent sniffing **(A)** conspecific and object in the social preference test, **(B)** familiar and novel conspecifics in the social recognition test, and **(C)** horizontal and **(D)** vertical locomotion during the social preference test. *p < 0.05, **p < 0.01 significant differences from a vehicle for the same target. ^#^*p* < 0.05, ^##^*p* < 0.01 significant differences between time spent sniffing both targets for the same dose of ethanol.

In this experiment, mice (*N* = 45) received saline or ethanol (0.25, 0.5, 1.0 or 1.5 g/kg) 10 min before been evaluated in the social preference test. The following day, the same animals were tested for social recognition memory in the absence of drug. Ethanol treatment, as shown by the one-way ANOVA, had a significant effect on time spent sniffing the conspecific (*F*_(4,40)_ = 20.12, *p* < 0.01), and planned comparisons revealed that ethanol at the lowest dose (0.25 g/kg) increased conspecific exploration (*p* < 0.01) in comparison with vehicle treatment, while higher doses decreased time with conspecific (1.0 and 1.5 g/kg, *p* < 0.05 and *p* < 0.01 respectively). The one-way ANOVA for time spent sniffing the object (*F*_(4,40)_ = 4.45, *p* < 0.01) was also significant. However, only the highest dose of ethanol (1.5 g/kg) significantly reduced (*p* < 0.01) time spent sniffing the object compared to the vehicle treated group (Figure 2A). When comparing time exploring both stimuli in the same animals, Student *t*-test for dependent samples showed that in the vehicle group there was a significant difference in time spent sniffing the conspecific vs. the object (*t* = −8.28, *p* < 0.01), a pattern that was repeated at all doses of ethanol (0.25 g/kg, *t* = −5.49, *p* < 0.01; 0.5 g/kg, *t* = −5.75, *p* < 0.01; 1.0 g/kg, *t* = 2.61, *p* < 0.05; 1.5 g/kg *t* = −2.76, *p* < 0.01; Figure 2A). Thus, independently of the ethanol dose used, all groups explored the conspecific more than the object, showing a clear preference for social interaction.

There was no significant effect of ethanol treatment on total crosses (*F*_(4,40)_ = 0.59, n.s.; Figure 2C) and on vertical locomotion (*F*_(4,40)_ = 2.25, n.s.; Figure 2D).

One day after the social interaction test took place, social recognition was evaluated, and the results of the one-way ANOVA showed an overall effect of previous exposure to ethanol on time spent sniffing the familiar conspecific (*F*_(4,40)_ = 2.08, *p* < 0.05). Ethanol at doses of 0.25 and 1.5 g/kg increased time spent at sniffing the familiar conspecific (*p* < 0.05 and *p* < 0.01 respectively) compared to the group previously treated with vehicle. A significant effect of ethanol administered the previous day was also observed on time spent sniffing the novel conspecific (*F*_(4,40)_ = 5.78, *p* < 0.01). Only animals that had received the lowest dose of ethanol (0.25 g/kg) increased time spent sniffing the novel conspecific in comparison with the vehicle group (*p* < 0.01; Figure 2B). Student’s *t*-test for dependent samples showed that vehicle animals spent more time sniffing the novel than the familiar conspecific (*t* = 5.32, *p* < 0.01), a pattern that was only observed in the group that had received the lower dose of ethanol (0.25 g/kg, *t* = 2.46, *p* < 0.05), suggesting that ethanol, even at doses that had no effect on social exploration the day before (0.5 g/kg), can impair social recognition 24 h after been administered.

### Experiment 2: Effect of The Non-Selective Adenosine A_1_/A_2A_ Antagonist Caffeine on Social Preference and Locomotion: Impact on Long-Term Social Recognition Memory

**Figure 3:**
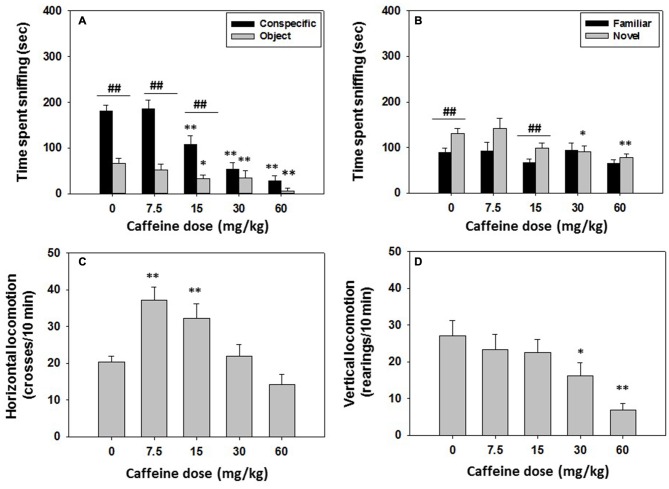
**Effect of caffeine on social preference and recognition tests.** Data are expressed as mean (±SEM) of time spent sniffing **(A)** conspecific and object in the social preference test, **(B)** familiar and novel conspecifics in the social recognition test, and **(C)** horizontal and **(D)** vertical locomotion during the social preference test. *p < 0.05, **p < 0.01 significant differences from a vehicle for the same target. ^##^*p* < 0.01 significant differences between time spent sniffing both targets for the same dose of caffeine.

Mice (*N* = 44) were injected with saline or caffeine (7.5, 15.0, 30.0 or 60.0 mg/kg) 30 min before the social interaction test started. The following day (24 h later) no drugs were administered and social recognition was evaluated as described before. The one-way ANOVA revealed an overall effect of caffeine on time spent sniffing the conspecific (*F*_(4,39)_ = 21.12, *p* < 0.01). Planned comparison analysis showed a significant decrement in time spent sniffing the conspecific after caffeine administration at doses of 15.0, 30.0 and 60.0 mg/kg (*p* < 0.01). The one-way ANOVA for the effect of caffeine on time spent sniffing the object (*F*_(3.39)_ = 4.03, *p* < 0.01) was also significant, and the planned comparisons revealed that the same doses of caffeine (15.0, 30.0 and 60.0 mg/kg) decreased time spent sniffing the object compared to vehicle (*p* < 0.05, *p* < 0.01 and *p* < 0.01, respectively). The Student’s *t*-test for dependent samples was used to compare time spent sniffing the conspecific with time spent sniffing the object. The vehicle treated group spent more time exploring the conspecific than the object (*t* = 5.24, *p* < 0.01), and this pattern of behavior was also preserved after the administration of moderate doses of caffeine (7.5 and 15.0 mg/kg; *t* = 6.28, *p* < 0.01, *t* = 3.84, *p* < 0.01 respectively) but not after the highest doses (30.0 and 60.0 mg/kg; Figure 3A), indicating a lack of preference for the conspecific after mice received the higher doses of caffeine.

The one-way ANOVA revealed an overall effect of caffeine on horizontal locomotion (*F*_(4,39)_ = 7.90 *p* < 0.01). Caffeine significantly increased horizontal locomotion at low to intermediate doses (7.5 and 15.0 mg/kg; *p* < 0.01) compared to vehicle, but did not have a significant effect at higher doses. The one-way ANOVA for vertical locomotion (*F*_(4,39)_ = 4.60 *p* < 0.01) was also significant, but for this dependent variable, planned comparisons revealed that the higher doses (30.0 and 60.0 mg/kg), significantly decreased vertical locomotion in comparison with the vehicle treated group (*p* < 0.05 and *p* < 0.01, respectively; Figures 3C,D). This decrease in locomotion could be influencing the reduction in time dedicated to targeted exploration, more importantly, to conspecific exploration.

For the social recognition results, the one-way ANOVA revealed no significant effect of the previous treatment with caffeine on time spent sniffing the familiar conspecific (*F*_(4,39)_ = 1.37, n.s.). However, there was an overall effect of previous caffeine treatment on time spent sniffing the novel conspecific (*F*_(4,39)_ = 3.83, *p* < 0.01). Planned comparisons revealed that compared with vehicle the highest doses of caffeine (30.0 and 60.0 mg/kg) significantly decreased time spent sniffing the novel conspecific (*p* < 0.05 and *p* < 0.01, respectively; Figure 3B). Student’s *t*-test for dependent samples showed that the vehicle group spent more time sniffing the novel conspecific than sniffing the familiar one (*t* = −3.40, *p* < 0.01), and this was also observed in the group that received 15.0 mg/kg of caffeine (*t* = −3.31, *p* < 0.01), but not the rest of the doses (Figure 3B).

### Experiment 3: Effect of Caffeine-Ethanol Co-Administration on Social Preference and Locomotion: Impact on Long-Term Social Recognition Memory

**Figure 4:**
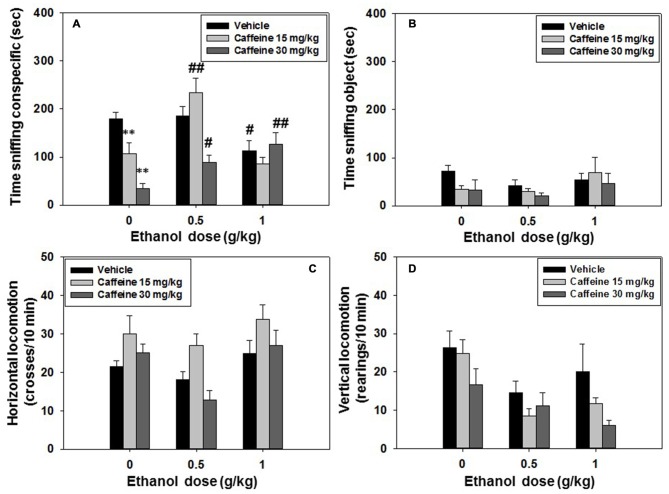
**Effect of caffeine plus ethanol interaction in the social preference test.** Data are expressed as mean (±SEM) of time spent sniffing **(A)** conspecific, **(B)** object, **(C)** horizontal and **(D)** vertical locomotion during the social preference test. *p < 0.05, **p < 0.01 significantly different from the vehicle group in the same dose of ethanol. ^#^*p* < 0.05, ^##^*p* < 0.01 significantly different from the group that received the same dose of caffeine plus ethanol 0.0 g/kg.

For experiment 3, mice (*N* = 74) received an injection of vehicle or caffeine (15.0 or 30.0 mg/kg; 30 min before being tested) plus vehicle or ethanol (0.5 or 1.0 g/kg; 10 min before test), and were evaluated for social preference and locomotion. The following day, the same animals were tested in the social recognition test. Factorial ANOVA (Caffeine × Ethanol) on time spent sniffing the conspecific showed overall effects of caffeine (*F*_(2,65)_ = 13.33, *p* < 0.01), and ethanol (*F*_(2,65)_ = 9.97, *p* < 0.01) and also a significant interaction (*F*_(4,65)_ = 8.99, *p* < 0.05). Planned comparisons confirmed that when compared with the vehicle-vehicle group only the highest dose of ethanol used in the present study (1.0 g/kg) reduced conspecific exploration (*p* < 0.05), and that the two doses of caffeine (15.0 and 30.0 mg/kg) selected for this experiment also reduced social exploration (*p* < 0.01). In terms of the interactions, the group that received the lowest dose of caffeine (15.0 mg/kg) in combination with the lowest dose of ethanol (0.5 g/kg) was significantly different (*p* < 0.01) from the group that had received that dose of caffeine but no ethanol, pointing to a reversal effect of ethanol on the caffeine-induced impairment. However, the effect of this dose of caffeine was not reversed when given in combination with the highest dose of ethanol (1.0 g/kg). As for the impairing effect on conspecific exploration observed in the group that had received the highest dose of caffeine (30.0 mg/kg) plus vehicle, this effect was partially reversed by the two doses of ethanol (*p* < 0.05 and *p* < 0.01 respectively; Figure 4A). The factorial ANOVA (Caffeine × Ethanol) for the dependent variable time spent sniffing, the conspecific did not show a significant effect of caffeine (*F*_(2,65)_ = 1.31, n.s.), of ethanol (*F*_(2,65)_ = 1.69, n.s.) or the interaction (*F*_(4,65)_ = 0.71, n.s.), (Figure 4B).

Factorial ANOVA (Caffeine × Ethanol) for total crosses as a measure of horizontal locomotion revealed an overall effect of caffeine (*F*_(2,65)_ = 7.22, *p* < 0.01), and ethanol (*F*_(2,65)_ = 6.27, *p* < 0.01), but no significant interaction (*F*_(4,65)_ = 0.77, n.s.), (Figure 4C). A separate factorial ANOVA for vertical locomotion showed the same pattern of results. It revealed an effect of caffeine (*F*_(2,65)_ = 4.23, *p* < 0.05), and of ethanol (*F*_(2,65)_ = 7.74, *p* < 0.01), but no significant caffeine-ethanol interaction (*F*_(4,65)_ = 0.81, n.s.; Figure 4D).

The results for the impact of these pharmacological manipulations on social recognition memory evaluated the day after the drug injection, and the preference test, are shown in Table 1. The factorial ANOVA (Caffeine × Ethanol) showed an overall effect of caffeine (*F*_(2,65)_ = 3.72, *p* < 0.05), and of ethanol (*F*_(2,65)_ = 8.27, *p* < 0.01) on time spent sniffing the familiar conspecific. However, there was no significant caffeine × ethanol interaction (*F*_(4,65)_ = 1.49, n.s.). In terms of time spent sniffing the novel conspecific, the factorial ANOVA revealed no significant effect of ethanol (*F*_(2,65)_ = 2.37, n.s.), but a significant effect of caffeine (*F*_(2,65)_ = 3.43, *p* < 0.05), and a significant interaction (*F*_(2,65)_ = 0.91, *p* < 0.01). The Student’s *t*-test for dependent samples comparing time spent sniffing familiar conspecific vs. novel conspecific revealed that the group that had received vehicle-vehicle injections the day before spent significantly more time sniffing the novel conspecific than the familiar conspecific (*t* = 4.96, *p* < 0.01), and the same was true for the animals treated with the low dose of caffeine (15.0 mg/kg) plus saline (*t* = 2.85, *p* < 0.05), indicating that mice recognized the familiar conspecific. However, the lower dose of caffeine (15.0 mg/kg) did not block the impairing effect on recognition produced by ethanol (0.5 or 1.0 g/kg).

**Table 1:** **Effect of caffeine-ethanol coadministration on social recognition memory**.

Etoh (g/kg)	0.0		0.5		1.0	
Caffeine (mg/kg)	Familiar	Novel	Familiar	Novel	Familiar	Novel
**0.0**	87.5 ± 9.1	136.4 ± 12.1^##^	111.4 ± 14.5	124.1 ± 16.6	115.9 ± 21.7	106 ± 19.1
**15.0**	71.2 ± 7.1	100.1 ± 13.2^#^	120.6 ± 27.3	98.1 ± 12.5	72.1 ± 10.5	102.3 ± 21.7
**30.0**	33.0 ± 11.1	31.9 ± 21.1	103.6 ± 11.8	137.2 ± 21.9	83.3 ± 11.2	91.1 ± 12.7

*Data are expressed as mean ± SEM of time (in seconds) spent sniffing the novel and the familiar conspecifics. ^##^*p* < 0.01, ^#^*p* < 0.05 significant differences between time with familiar vs. time with novel conspecific for the same dose of ethanol group*.

### Experiment 4: Effect of The Selective Adenosine A_1_ Receptor Antagonist CPT on Social Preference and Locomotion: Impact on Long-Term Social Recognition Memory

**Figure 5:**
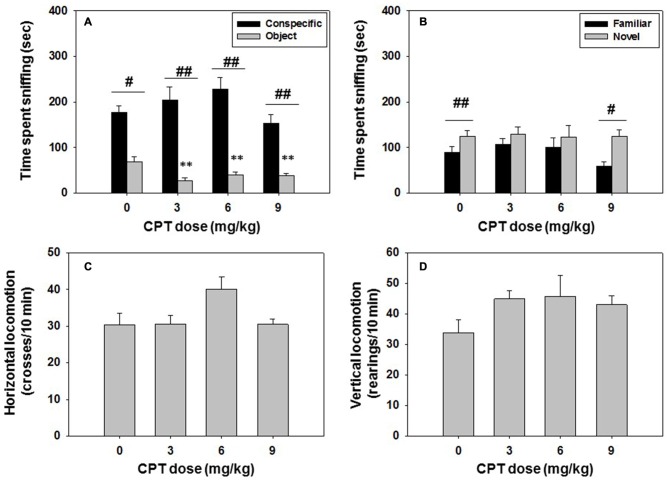
**Effect of Cyclopentyltheophylline (CPT) in the social preference and recognition tests. (A)** Conspecific and object in the social preference test, **(B)** familiar and novel conspecifics in the social recognition test, and **(C)** horizontal and **(D)** vertical locomotion during the social preference test. **p < 0.01 significant differences from vehicle for the same target. ^#^*p* < 0.05, ^##^*p* < 0.01 significant differences between time spent sniffing both targets for the same dose of CPT.

Mice (*N* = 37) were injected with vehicle or CPT at doses of 3.0, 6.0, or 9.0 mg/kg 30 min before being tested in the social preference task, and 24 h later the same animals were tested in the social recognition test. The effect of CPT on time spent sniffing the conspecific analyzed by a one-way ANOVA revealed no significant effect (*F*_(3,33)_ = 2.13, n.s.). However, the one-way ANOVA on the effect of CPT on time spent sniffing the non-social target was significant (*F*_(3,33)_ = 5.21, *p* < 0.01). Planned comparison revealed that CPT significantly decreased time spent exploring the object at all doses of CPT in comparison with the vehicle group (*p* < 0.01; Figure 5A), suggesting an increase in relative preference for the conspecific. Student’s *t-test* for dependent samples showed significant differences in all the groups in time spent sniffing the conspecific vs. the object. Animals spent more time sniffing the conspecific after saline (*t* = 5.37, *p* < 0.05), CPT 3.0 mg/kg (*t* = 11.25, *p* < 0.01), CPT 6.0 mg/kg (*t* = 6.38, *p* < 0.01), and CPT 9.0 mg/kg (*t* = 5.95, *p* < 0.01).

These doses of CPT did not affect the horizontal (*F*_(3,33)_ = 1.03, n.s.) or vertical locomotion (*F*_(3,33)_ = 1.42, n.s.), as analyzed by one-way ANOVA’s (Figures 5C,D).

For the social recognition test, the one-way ANOVA did not show a significant effect of CPT dose on time spent sniffing the familiar conspecific (*F*_(3,33)_ = 0.14, n.s.), or on time spent sniffing the novel conspecific (*F*_(3,33)_ = 0.02, n.s.). Student’s *t*-test for dependent samples showed significant differences between time spent sniffing the novel vs. the familiar conspecific in the vehicle group (*t* = −3.82, *p* < 0.01), as expected when animals recognized the previously explored conspecific, and this effect was also observed in the animals that had received the highest dose of CPT 9.0 mg/kg the day before (*t* = −3.25, *p* < 0.05), but not the lower doses (Figure 5B).

### Experiment 5: Effect of CPT–Ethanol Co-Administration on Social Preference and Locomotion: Impact on Long-Term Social Recognition Memory

**Figure 6:**
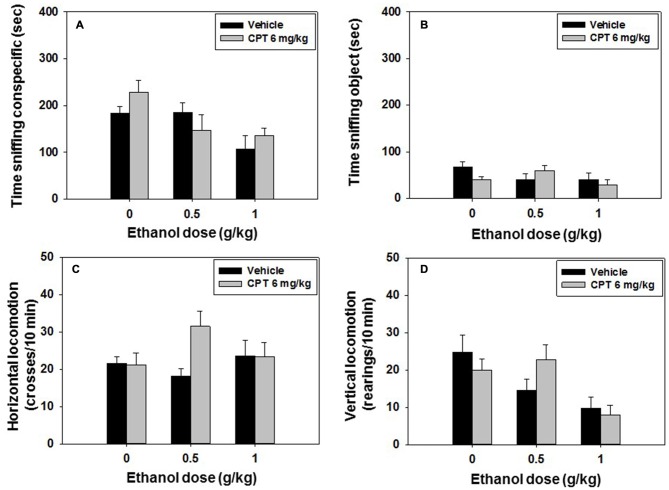
**Effect of CPT plus ethanol interaction on the social preference test.** Data are expressed as mean (±SEM) of time spent sniffing **(A)** conspecific, **(B)** object, **(C)** horizontal and **(D)** vertical locomotion during the social preference test.

Mice (*N* = 60) received an injection of vehicle or CPT 6.0 mg/kg 20 min before the test, and a second injection of vehicle or ethanol (0.5 or 1.0 g/kg) 10 min before the social preference test started. The following day, the same animals were tested in the social recognition test with no drug been administered. A factorial ANOVA (CPT × Ethanol) showed an overall effect of ethanol (*F*_(2,41)_ = 5.33, *p* < 0.05), but no significant effect of CPT (*F*_(1,41)_ = 0.32, n.s) or CPT-ethanol interaction (*F*_(2,41)_ = 1.60, n.s.) on time spent sniffing the conspecific (Figure 6A). The factorial ANOVA for time spent sniffing the object (Figure 6B) did not reveal a significant effect of CPT (*F*_(1,41)_ = 0.43, n.s.), of ethanol (*F*_(2,41)_ = 1.46, n.s.), or of the interaction (*F*_(2,41)_ = 2.21, n.s.) either.

The factorial ANOVA (CPT × Ethanol) on horizontal locomotion yield no significant effect of ethanol (*F*_(2,41)_ = 0.55, n.s.), CPT (*F*_(1,42)_ = 2.36, n.s) or CPT-ethanol interaction (*F*_(2,41)_ = 2.86, n.s.; Figure 6C). As for vertical locomotion, there was a significant effect of ethanol (*F*_(2,41)_ = 6.59, *p* < 0.01), but not a significant effect of CPT (*F*_(1,41)_ = 0.03, n.s.) or of CPT-ethanol interaction (*F*_(2,41)_ = 1.82, n.s.; Figure 6D).

For the social recognition test the factorial ANOVA (CPT × Ethanol) did not show a significant effect of CPT (*F*_(1,41)_ = 1.06, n.s.), of ethanol (*F*_(2,41)_ = 0.97, n.s.), or of the interaction (*F*_(2,41)_ = 0.05, n.s.) on time spent sniffing the familiar conspecific. The factorial ANOVA for the variable time spent sniffing the novel conspecific, did not show an overall effect of CPT (*F*_(1,41)_ = 0.38, n.s), ethanol (*F*_(2,41)_ = 1.78, n.s.), or CPT-ethanol interaction (*F*_(2,41)_ = 1.11, n.s.) either. Student’s *t*-test for dependent samples showed significant differences between time spent at sniffing the novel vs. familiar conspecific only in the control group (*t* = 4.7, *p* < 0.01), confirming that ethanol as shown before impaired social recognition at all doses, and indicating that CPT (6 mg/kg) did not block the amnestic effects of ethanol (data shown in Table 2).

**Table 2:** **Effects of Cyclopentyltheophylline (CPT) ethanol combination on social recognition memory**.

Etoh (g/kg)	0.0		0.5		1.0	
CPT (mg/kg)	Familiar	Novel	Familiar	Novel	Familiar	Novel
**0.0**	74.1 ± 4.5	139.4 ± 12.4^##^	122.6 ± 12.4	124.1 ± 16.6	99.0 ± 22.0	102.5 ± 12.4
**6.0**	100.1 ± 20.5	123.5 ± 25.3	111.0 ± 14.6	172.3 ± 27.8	119.6 ± 23.3	105.0 ± 27.8

*Data are expressed as mean ± SEM of time in seconds spent sniffing novel and familiar conspecifics. ^##^*p* < 0.01 significant differences between time in familiar vs. time in novel conspecific for the same dose of CPT and ethanol*.

### Experiment 6: Effect of The Selective Adenosine A_2A_ Receptor Antagonist MSX-3 on Social Preference and Locomotion: Impact on Long-Term Social Recognition Memory

**Figure 7:**
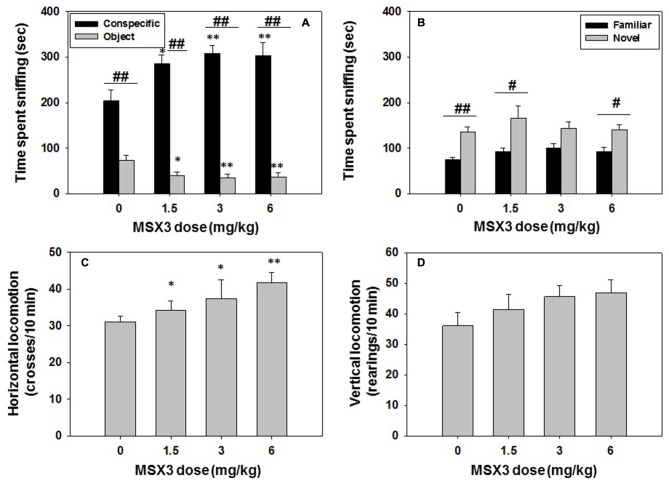
**Effect of MSX3 in social preference and recognition tests.** Data are expressed as mean (±SEM) of time spent sniffing **(A)** conspecific and object in the social preference test, **(B)** familiar and novel conspecifics in the social recognition test, and **(C)** horizontal and **(D)** vertical locomotion during the social preference test. *p < 0.05, **p < 0.01 significant differences from vehicle for the same target. ^#^*p* < 0.05, ^##^*p* < 0.01 significant differences between time spent sniffing both targets for the same dose of MSX3.

Different groups of mice (*N* = 36) received an acute administration of vehicle or MSX-3 at doses of 1.5, 3.0, or 6.0 mg/kg, 30 min before the social interaction test. The same animals were tested 24 h later in the social recognition test. The one-way ANOVA revealed an overall effect of MSX-3 on time spent sniffing the conspecific (*F*_(3,32)_ = 4.58, *p* < 0.01), and planned comparison showed that all doses increased significantly the time spent sniffing the social target (1.5 mg/kg, *p* < 0.05; 3.0 mg/kg and 6.0 mg/kg, *p* < 0.01) compared with the vehicle treated group. The one-way ANOVA for the dependent variable time spent exploring the object was also significant (*F*_(3,32)_ = 3.63, *p* < 0.05). MSX-3 significantly decreased the time exploring the object at all doses (1.5 mg/kg, *p* < 0.05; 3.0 mg/kg and 6.0 mg/kg, *p* < 0.01) when compared with the vehicle group. Student *t*-test for dependent samples demonstrated that there were significant differences in time spent sniffing the conspecific vs. the object in the vehicle group (*t* = 12.96, *p* < 0.01), but also in all the MSX-3 treated groups (MSX-3 1.5 mg/kg, *t* = 7.96, *p* < 0.01; MSX-3 3.0 mg/kg, *t* = 10.33 *p* < 0.01, and MSX-3 6.0 mg/kg, *t* = 6.87 *p* < 0.01; Figure 7A).

The impact of MSX-3 on locomotion is shown in Figures 7C,D. The ANOVA for the effect of MSX-3 on horizontal locomotion was significant (*F*_(3,32)_ = 3.66, *p* < 0.05), and planned comparisons showed a significant effect of all doses of MSX-3 on total crosses between compartments as a measure of horizontal locomotion (1.5 mg/kg and 3.0 mg/kg, *p* < 0.05; and 6.0 mg/kg, *p* < 0.01). However, the one-way ANOVA for vertical locomotion was not significant (*F*_(3,32)_ = 1.83, n.s.).

For the social recognition test, the one-way ANOVA revealed no significant effect of MSX-3 on time spent sniffing the familiar conspecific (*F*_(3,32)_ = 1.83, n.s.), and also no significant effect of this drug on novel conspecific exploration (*F*_(3,32)_ = 0.61, n.s.; Figure 7B). Student’s *t*-test for dependent samples showed significant differences between time spent sniffing novel vs. familiar conspecific in the vehicle group (*t* = −4.71, *p* < 0.01), as expected, and this pattern was also observed in the MSX-3 1.5 mg/kg, (*t* = −2.64, *p* < 0.05) and the MSX-3 6.0 mg/kg groups (*t* = −2.42, *p* < 0.05). The intermediate dose of MSX-3 3.0 mg/kg almost reach significant levels (*t* = −2.13, *p* = 0.06). Thus, MSX-3 administered the day before did not affect social recognition memory.

### Experiment 7: Effect of MSX3–Ethanol Co-Administration on Social Preference and Locomotion: Impact on Long-Term Social Recognition Memory

**Figure 8:**
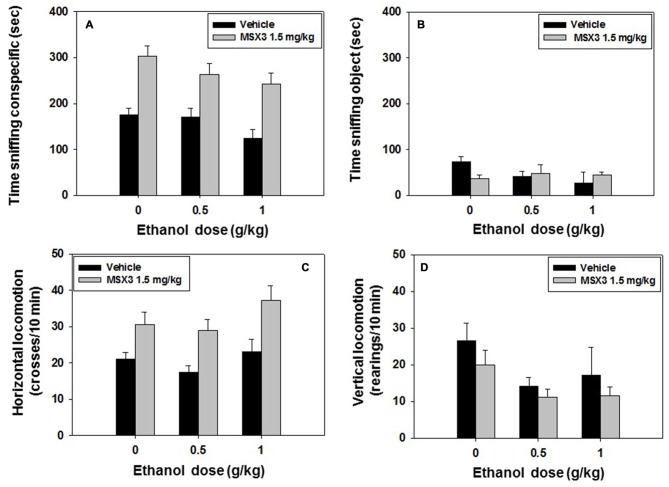
**Effect of MSX3 plus ethanol interaction in the social preference test.** Data are expressed as mean (±SEM) of time spent sniffing **(A)** conspecific, **(B)** object, **(C)** horizontal and **(D)** vertical locomotion during the social preference test.

Mice (*N* = 50) received a dose of vehicle or of the lowest dose of MSX-3 (1.5 mg/kg) that was effective in experiment 6. MSX-3 was administered 20 min before test, and 10 min before the social preference test, a second injection of vehicle or ethanol (0.5 or 1.0 g/kg) was administered. The following day, the same animals were tested for social long-term memory. A factorial ANOVA (MSX-3 × Ethanol) revealed an overall effect of MSX-3 (*F*_(1,43)_ = 40.65, *p* < 0.01), and of ethanol (*F*_(2,43)_ = 3.36, *p* < 0.05) on time spent sniffing the conspecific. However, there was not a significant interaction (*F*_(2,43)_ = 0.34, n.s.; Figure 8A). The factorial ANOVA for time spent sniffing the object did not reveal a significant effect of MSX-3 (*F*_(1,43)_ = 1.45, n.s.), or ethanol (*F*_(2,43)_ = 0.49, n.s.), and no significant interaction (*F*_(2,43)_ = 2.23, n.s.) either (Figure 8B).

Total crosses between compartments as a measure of horizontal locomotion were overall affected by MSX-3 (*F*_(1,43)_ = 21.18, *p* < 0.01), but not by ethanol (*F*_(2,43)_ = 2.42, n.s.), and there was no significant interaction either (*F*_(2,43)_ = 0.30, n.s.). The one-way ANOVA for vertical locomotion revealed a significant effect of ethanol (*F*_(2,43)_ = 3.99, *p* < 0.05), but no effect of MSX3 (*F*_(1,43)_ = 2.27, n.s.), and no significant interaction (*F*_(2,43)_ = 0.11, n.s; See Figures 8C,D).

As for the impact of these drugs on recognition of the conspecific presented during the preference test, the factorial ANOVA (MSX-3 × Ethanol) for time spent sniffing the familiar conspecific showed a significant effect of ethanol (*F*_(2,43)_ = 6.97, *p* < 0.01), but did not show an effect of MSX-3 (*F*_(1,43)_ = 0.02, n.s.), and no MSX-3 × ethanol interaction on this variable (*F*_(2,43)_ = 2.14, n.s.; Table 3). Another factorial ANOVA for the variable time spent sniffing the novel conspecific, did not reveal an effect of MSX-3 (*F*_(1,43)_ = 0.14, n.s.), it did not show a significant effect of ethanol although it was close to significance (*F*_(2,43)_ = 2.73, *p* = 0.08), and the interaction was not significant (*F*_(2,43)_ = 0.43, n.s.). When comparing the behavior of every group of animals in the exploration of the known and novel conspecific, the control group that had been treated with vehicle-vehicle the day before spent significantly more time sniffing the novel conspecific vs. the familiar conspecific as expected if the animal recognizes the known conspecific (*t* = 4.71, *p* < 0.01). This result was also observed in animals treated with MSX-3 1.5 mg/kg plus vehicle (*t* = 2.64, *p* < 0.05). As expected, animals treated with vehicle plus ethanol (at either dose) did not recognize the familiar animal and explored both conspecific equally. However, MSX-3 1.5 mg/kg blocked the effect of the lowest dose of ethanol 0.5 g/kg (*t* = 2.52, *p* < 0.05), although not the highest dose of ethanol. Thus, it seems that MSX-3 had a preventive effect only when the dose of ethanol was low.

**Table 3:** **Effects of MSX3-ethanol combination on social recognition memory**.

Etoh (g/kg)	0.0		0.5		1.0	
MSX3 (mg/kg)	Familiar	Novel	Familiar	Novel	Familiar	Novel
**0.0**	75.1 ± 4.9	138.3 ± 14.0^##^	105.6 ± 11.9	118.7 ± 13.3	120.0 ± 24.6	114.6 ± 19.8
**1.5**	84.1 ± 10.9	160.1 ± 24.8^#^	68.7 ± 7.8	109.3 ± 12.3^#^	142.6 ± 20.0	117.8 ± 12.2

*Data are expressed as mean ± SEM of time in seconds spent sniffing novel or familiar conspecifics. ^##^*p* < 0.01, ^#^*p* < 0.05 significant differences between time in familiar vs. time in novel conspecific for the same dose of MSX3 and ethanol*.

## Discussion

In the present study, we characterize the impact of two of the most commonly consumed drugs of abuse, caffeine and alcohol, on motivation for social contact as manifested by social preference or avoidance, and also on consolidation of social memories. We evaluated the possibility of a common mechanism of action for both drugs via the adenosine system. Thus, we hypothesized that low to intermediate doses of alcohol could lead to an increase in adenosine levels that would counteract the effect of caffeine, which acts as a non-selective A_1_ and A_2A_ antagonist. In order to test that hypothesis, the effects of selective A_1_ and A_2A_ receptor antagonists were also assessed alone or in combination with ethanol.

Our results show that the suppressing effects of high doses of caffeine on social approach and preference can be counteracted by low doses of ethanol, but this reversal effect reaches a ceiling when ethanol starts to mildly impair social approach and preference on its own. Social interaction has been mostly used to evaluate anxiety in rodents, because it was found that anxiolytics increase time spent in active social interaction while anxiogenic drugs decrease social contact independently of any change in activity (File and Hyde, 1978; Guy and Gardner, 1985). Thus, the reduction in social preference observed after caffeine administration could be explained by an increase in anxiety, since doses ranging from 25.0 to 100.0 mg/kg have been demonstrated to have a substantial anxiogenic effect in this strain of mice as seen in the elevated plus maze (López-Cruz et al., 2013). It is also possible that anxiolysis induced by ethanol could be playing a role in potentiating social interaction as suggested by previous researchers (Hilakivi et al., 1989; Nadal et al., 1993). However, it cannot be the only explanation for this effect since doses of ethanol that induced anxiolysis in this strain of mice (0.5 and 1.0 g/kg) in an elevated plus maze (Correa et al., 2008) were not able to reverse social preferences to normal levels. Moreover, in the present study we used a procedure developed to minimize anxiety in the experimental mouse by eliminating the possibility of physical aggression since the target mouse was enclosed in a wire cage (Crawley, 2004; Moy et al., 2004). Thus, in this paradigm it is possible to assess preference or avoidance for social interaction based on free choice. Furthermore, none of the pharmacological manipulations used in the present series of studies produced a significant avoidance for the compartment where the conspecific was located (data not shown). The effects of caffeine and ethanol alone or in combination on social behavior do not seem to be mediated by their effects on locomotion either, because the range of doses used do not clearly impair locomotion, and an increase in locomotion induced by the lowest doses of caffeine (7.5 and 15.0 mg/kg) seems to be unrelated to social exploration.

Although a strength of the present study was the use of a broad range of doses for all drugs, including the studies of drug interaction (most of the previous studies have used a single dose approach), it is not clear that the effect of high doses of caffeine were mediated by its actions on adenosine A_1_ and A_2A_ receptors, since neither of the selective adenosine receptors reduced social interaction at the doses tested. Because in the present paradigm the experimental mouse has to explore a broad area that separates the two targets (conspecific and object), we selected doses of caffeine and selective adenosine antagonists based on results from previous work showing no impairing effects on ambulation and rearing in an open field (Pardo et al., 2013; López-Cruz et al., 2014), in order to avoid the possibility of mediating variables related to motor function. Thus, the A_1_ antagonist CPT did not produce a significant change in social approach and preference, although mice spent more time in the conspecific compartment at the low doses (data not shown), and there was no interaction with ethanol on these parameters. It is possible, however, that higher doses of CPT could mimic the effects of caffeine on social preference, specially taking into account that previous studies have demonstrated that caffeine, at the same dose used in the present study (30.0 mg/kg), and the A_1_ antagonist DPCPX produced an anxiogenic-like effect in mice, and reversed ethanol anxiolytic actions (Prediger et al., 2004). On the other hand, the A_2A_ receptor antagonist MSX-3 did have a significant effect, increasing preference for the social target and reducing it for the object. It is also worth noting that although general exploration (crossings between the three compartments) increased, MSX-3 did not disturb focused social exploration. Moreover, there was no significant interaction between MSX-3 and ethanol on any of these parameters; the improving effect of MSX-3 on preference was maintained at the same level independently of the dose of ethanol (0.5 or 1.0 g/kg) that the animals received. Consistently, high levels of social interaction have been observed in A_2A_ receptor KO mice, and these animals were not affected by a dose of ethanol (1.0 g/kg) that impaired social interaction (López-Cruz et al., in press). Interestingly, A_2A_KO mice showed an anxiogenic profile, which again argues against a straight relationship between anxiety and social interaction (López-Cruz et al., in press).

A decrease in exploring a familiar conspecific when a new one is also present has been interpreted as an index of social recognition (Thor and Holloway, 1982; Crawley, 2004; Moy et al., 2004), which some authors consider to be also an index of preference for novelty seeking (Costa et al., 2014). Whatever the interpretation, it is required that the animal consolidates a memory for the familiar conspecific. Adenosine seems to modulate short-term social memory in rats by acting on both A_1_ and A_2A_ receptors, with adenosine receptor agonists and antagonists respectively disrupting and enhancing social recognition memory (Prediger and Takahashi, 2005). Thus, the selective A_1_ agonist CCPA and the A_2A_ agonist DPMA disrupted juvenile recognition in adult rats (Prediger and Takahashi, 2005). This impairment of short-term social memory induced by adenosine agonists was reversed by caffeine, the A_1_ antagonist DPCPX, and the A_2A_ antagonist ZM24138 (Prediger and Takahashi, 2005). Moreover, acute administration of caffeine or selective A_2A_ antagonists reversed the disruption of social recognition memory in ageing rats (Prediger et al., 2005a), and also in spontaneously hypertensive rats (Prediger et al., 2005b) in which some alterations in adenosine neurotransmission have been reported (Davies et al., 1987; Matias et al., 1993; Lopes et al., 1999). However, all these studies evaluated short-term social memory and not long-term social memory. If the recognition test is carried 24 h after the first presentation it can be considered as a test of long-term memory processes. The development and consolidation of long-term potentiation seems to be also modulated by adenosine receptor-dependent mechanisms in the hippocampus (Tanaka et al., 1990; de Mendonca and Ribeiro, 1994; Hauber and Bareiss, 2001). Data from the present study indicates that caffeine at high doses impaired recognition on the following day, especially at those doses (30.0 and 60.0 mg/kg) that had reduced relative preference for social interaction the day before. Thus, mice explored familiar and novel conspecifics equally, which could be explained by the fact that animals had explored the conspecific for much less time the day before than animals under control conditions. It is possible that the ability of caffeine to improve memory at low doses could be seen under different experimental conditions. In fact, theophylline (another non-selective A_1_/A_2A_ antagonist) has been shown to facilitate long-term spatial reference memory in retention sessions, but not in working memory, both of which are tasks that are highly dependent on hippocampus (Hauber and Bareiss, 2001). Thus, when the nature of the task involves optimal performance during basal conditions, it is very difficult to improve performance.

It is well known that ethanol can produce amnestic effects and impair retrieval of memories after the drug wears off (Goodwin, 1995; Hartzler and Fromme, 2003; Gulick and Gould, 2007, 2009). Ethanol-induced memory impairments can be produced by disruption of attention, and also by affecting neural mechanisms involved in memory consolidation such as the adenosinergic system (Tanaka et al., 1990; Gulick and Gould, 2007, 2009). In experiment 1, ethanol, even at doses that did not impair social interaction (0.5 g/kg), impaired social recognition 24 h later. Although this situation was characterized by low performance, caffeine (15.0 or 30.0 mg/kg) co-administration was not able to block the amnestic effects of ethanol. A previous study in rats explored the effect of caffeine-ethanol interaction on long-term memory using social odors (Spinetta et al., 2008). In that study ethanol was administered immediately after exposure to the social odor, and a recognition test was performed 24 h later (Spinetta et al., 2008). Caffeine, at a low dose that did not have an effect on its own (5.0 mg/kg), was able to prevent the disruptive effects of ethanol (1.0 g/kg) on memory consolidation (Spinetta et al., 2008). It is possible that in our study lower doses of caffeine could have improved ethanol-induced deficits. The behavioral effects induced by methylxantines at low doses are likely to be mediated by nonselective adenosine A_1_/A_2A_ receptor blockade, while higher doses might involve additional mechanisms such as inhibition of phosphodiesterases (Nehlig et al., 1992; Hauber and Bareiss, 2001).

As for the role of selective adenosine receptor antagonists, it appears that although CPT did not affect social interaction, it mildly impaired long-term social recognition at low doses, an effect that was not observed at high doses. CPT was not able to reverse the ethanol-induced impairment of recognition memory. In contrast, the selective A_2A_ antagonist MSX-3, which increased preference for the conspecific when administered alone, did not impair social recognition, and was able to block the amnestic effect of the lower dose of ethanol (0.5 g/kg). Thus, in our studies a selective A_2A_ antagonist was able to improve social memory under conditions of suboptimal performance (ethanol amnestic effects), but not under optimal performance (i.e., non-treated animals). This improvement in memory might be due to actions on processes involved in learning, such as attention and wakefulness, but may also be related to direct actions on memory systems. Alternatively, it is possible that MSX3 blocks ethanol’s amnestic effects because it robustly increases active sniffing of the conspecific. It has been demonstrated that sensory impoverishment in rats (by whisker clipping) exacerbates ethanol-induced deficits in social interaction (Wellmann and Mooney, 2015). Thus, under different experimental conditions that promote sensory exploration (such as sniffing behavior), it could be possible that ethanol’s amnestic effects would be diminished.

Although it is clear that normal social interaction is required for normal retrieval of social memories, the data from the present studies indicate a relative independence between social preference and social long-term memory processes. The results available at the present moment also suggest that A_1_ receptors do not seem to regulate social motivation and social recognition, since blocking their tonic activity has very little effect. A_1_ receptor antagonists appear to play only a modest role in the regulation of dopamine-dependent aspects of motivated behaviors (Pardo et al., 2012; Salamone and Correa, 2012). A_2A_ antagonists have similar motivational effects to dopamine uptake inhibitors (Yohn et al., 2016a,b), and since A_2A_ receptors are densely localized in dopamine rich areas such as the nucleus accumbens (Fredholm et al., 2001), it is possible that the modulation provided by A_2A_ antagonists on ethanol effects could be the result of a potentiation of the motivational functions regulated by this nucleus. Moreover, because selective A_1_ and A_2A_ antagonists did not mimic the effects of caffeine, it is possible that blockade of both receptors is necessary for producing a caffeine-like action. Alternatively, it is possible that at high doses caffeine may not be acting solely as an adenosine antagonist. Thus, although an increase in adenosine levels could be mediating ethanol effects, the usefulness of highly caffeinated drinks in counteracting ethanol-induced impairments on these normal social processes is questionable.

## Author Contributions

LL-C conducted the experiments, analyzed the data and wrote the draft of the manuscript, NS-M and PB conducted part of the experiments and helped in the evaluation of the variables. YB and CEM provided information and synthesized the drug MSX3. JDS and MC were involved in the design of experiments, and writing of the manuscript.

## Conflict of Interest Statement

The authors declare that the research was conducted in the absence of any commercial or financial relationships that could be construed as a potential conflict of interest.
